# Deletion of alpha-synuclein decreases impulsivity in mice

**DOI:** 10.1111/j.1601-183X.2011.00758.x

**Published:** 2011-11-16

**Authors:** Y Peña-Oliver, V L Buchman, J W Dalley, T W Robbins, G Schumann, T L Ripley, S L King, D N Stephens

**Affiliations:** † School of Psychology, University of SussexFalmer, Brighton BN1 9QG, UK; ‡ School of Biosciences, Cardiff UniversityMuseum Avenue, Cardiff CF10 3AX, UK; § Behavioural and Clinical Neuroscience Institute and Department of Experimental Psychology, University of CambridgeDowning Street, Cambridge CB2 3EB, UK; ¶ Department of Psychiatry, Addenbrooke's Hospital, University of CambridgeHill's Road, Cambridge CB2 2QQ, UK; ** Institute of Psychiatry, Kings CollegeDenmark Hill, London, SE5 8AF

**Keywords:** Alpha-synuclein, impulse control disorders, impulsivity, Parkinson's disease

## Abstract

The presynaptic protein alpha-synuclein, associated with Parkinson's Disease (PD), plays a role in dopaminergic neurotransmission and is implicated in impulse control disorders (ICDs) such as drug addiction. In this study we investigated a potential causal relationship between alpha-synuclein and impulsivity, by evaluating differences in motor impulsivity in the 5-choice serial reaction time task (5-CSRTT) in strains of mice that differ in the expression of the alpha-synuclein gene. C57BL/6JOlaHsd mice differ from their C57BL/6J ancestors in possessing a chromosomal deletion resulting in the loss of two genes, snca, encoding alpha-synuclein, and mmrn1, encoding multimerin-1. C57BL/6J mice displayed higher impulsivity (more premature responding) than C57BL/6JOlaHsd mice when the pre-stimulus waiting interval was increased in the 5-CSRTT. In order to ensure that the reduced impulsivity was indeed related to *snca*, and not adjacent gene deletion, wild type (WT) and mice with targeted deletion of alpha-synuclein (KO) were tested in the 5-CSRTT. Similarly, WT mice were more impulsive than mice with targeted deletion of alpha-synuclein. Interrogation of our ongoing analysis of impulsivity in BXD recombinant inbred mouse lines revealed an association of impulsive responding with levels of alpha-synuclein expression in hippocampus. Expression of beta- and gamma-synuclein, members of the synuclein family that may substitute for alpha-synuclein following its deletion, revealed no differential compensations among the mouse strains. These findings suggest that alpha-synuclein may contribute to impulsivity and potentially, to ICDs which arise in some PD patients treated with dopaminergic medication.

Alpha-synuclein has received increasing interest because of its role in Parkinson's disease (PD) (Chartier-Harlin *et al.* 2004; Kruger *et al.* 1998; Singleton *et al.* 2003; Spillantini *et al.* 1998), in which mutations and haplotypes of the *snca* gene encoding alpha-synuclein are associated with development of, or increased risk for the disease (Kruger *et al.* 1998; Polymeropoulos *et al.* 1997; Satake *et al.* 2009; Zarranz *et al.* 2004). A premorbid low impulsive personality in PD patients (Menza 2000; Todes & Lees 1985), characterized by ‘caution, constraint and low novelty-seeking’ has been suggested. Conversely, some PD patients maintained on dopaminergic drugs develop impulse control disorders (ICDs) and uncontrolled drug use (Evans *et al.* 2009; Voon *et al.* 2009).

Although the normal biological functions of alpha-synuclein are not well understood, it is postulated to play a role in synaptic vesicular transport and synaptic plasticity (Nemani *et al.* 2010). Mice lacking alpha-synuclein show alterations in dopaminergic neurotransmission in striatal areas (Abeliovich *et al.* 2000; Anwar *et al.* 2011). Furthermore, a growing literature also suggests a role for alpha-synuclein in addiction (Boyer & Dreyer 2007); expression of alpha-synuclein is increased in brain reward areas of cocaine addicts (Mash *et al.* 2003; Qin *et al.* 2005) and following psychostimulant treatment in rodents (Brenz Verca *et al.* 2003; Fornai *et al.* 2005). The role of impulsivity in drug addiction is increasingly recognized (Dawe & Loxton 2004; Perry & Carroll 2008), and addictive behaviour may be viewed as an example of impaired impulse control (Ambermoon *et al.* 2010).

One form of impulsivity refers to the difficulty in inhibiting incorrect or inappropriate responses – motor (Evenden 1999; Robbins 2002) or waiting impulsivity (Robinson *et al.* 2009) – which can be assessed in rodents with the 5-CSRTT (Carli *et al.* 1983; Oliver *et al.* 2009; Robbins 2002; Walker *et al.* 2011). This task assesses attentional performance by the detection of brief visual stimulus presented pseudo-randomly across five spatial locations, requiring a nose-poke into the correct location to obtain a reward. The 5-CSRTT also provides information about aspects of inhibitory response control: premature responding into the holes provides a measure of impulsivity (Robbins 2002); perseverative responding after correct detections represents a measure of compulsivity (Dalley *et al.* 2011). In order to investigate a potential relationship between alpha-synuclein and impulsivity, we evaluated motor impulsivity in strains of mice that differ in the expression of alpha-synuclein. In the first experiment, we compared C57BL/6JOlaHsd mice, that display a spontaneous chromosomal deletion leading to the loss of two genes, the snca gene encoding alpha-synuclein, and the mmrn1, encoding multimerin-1 (Specht & Schoepfer 2001, 2004), with their C57BL/6J ancestral strain. Although *mmrn1* has not been shown to be expressed in the brain (Specht & Schoepfer 2004), and is therefore unlikely to contribute to the behavioural phenotype of C57BL/6JOlaHsd mice, in order to ensure that the differences in impulsivity were indeed related to *snca*, and not adjacent genes affected by the chromosomal deletion, a second experiment was carried out in which wild type (WT) and mice with targeted deletion of alpha-synuclein (KO) were tested in the 5-CSRTT. Finally, we correlated levels of snca expression with impulsivity in a panel of BXD recombinant inbred mouse strains (Chesler *et al.* 2003).

## Materials and methods

### Subjects

For Experiment 1, male mice were obtained from Charles River Laboratories (Arbresle, France) (C57BL/6J; C; *n* = 12) and from Harlan Laboratories (Bicester, Oxon, UK) (C57BL/6JOlaHsd; H; *n* = 12).

For Experiment 2 mice carrying targeted null mutation of the gene encoding alpha-synuclein (Abeliovich *et al.* 2000) were produced in Cardiff University Transgenic Animal Unit by 11 generations of backcrosses with C57BL/6J mice (Charles River). At this stage heterozygous animals were intercrossed and the resulting WT and homozygous null mutant animals were used as breeders for production of experimental cohorts. Thus, experimental mice were the F2 offspring of homozygous knock-out (KO) or WT parents obtained by breeding heterozygous parents. Mouse genotyping was carried out as described previously (Robertson *et al.* 2004). A total of 31 (15 WT and 16 KO) male mice were shipped to the University of Sussex for testing.

Experiment 3 used male mice from the BXD series of recombinant inbred strains, purchased from Jackson Laboratories (Bar Harbor, ME, USA). In addition to the C57BL/6J and DBA2/J parent lines, the following lines were used: BXD5, BXD11, BXD12, BXD18, BXD21, BXD29, BXD31, BXD32, BXD33 and BXD36. Eight male mice from each strain were imported, and accustomed to the University of Sussex facility for at least 1 month before testing in the 5-CSRTT. Since we found strain BXD11 to be highly aggressive, it was necessary to house this strain in individual cages. In order to maintain similar conditions across groups, all other strains were held under identical conditions. All mice (from Experiments 1, 2 and 3) were 8–12-weeks old at the start of 5-CSRTT training.

Mice were singly-housed, had free access to food and water and were maintained on a 12 h light/dark cycle (lights off at 1900 h) at a temperature of 19–21°C and 50% humidity. Mice were food restricted to reduce their body weights to 85% of their free-feeding weight. A total of five animals did not complete the 5-CSRTT experiment, one mouse from the C group and one mouse from the H group died during the experiment and their data for the 5-CSRTT were not included in the analysis. Two C animals and one H animal failed to learn the 5-CSRTT and were excluded from the experiment. All experiments were approved by the institutional ethics committee and were performed under United Kingdom legislation on animal experimentation [Animal (Scientific Procedures) Act, 1986].

### Five-choice serial reaction time task apparatus

The test apparatus consisted of eight mouse operant chambers (Med Associates Inc., St. Albans, VT, USA). Each chamber was housed in a sound-attenuating outer cabinet, with a ventilator fan providing a constant low-level background noise. The left wall of the chamber was curved and contained five apertures fitted with infrared detectors to detect nose-poke responses. The apertures were illuminated by a yellow stimulus light located inside each aperture. The right wall of the chamber contained a receptacle hole with a round access opening where the liquid reinforcer was delivered. Condensed milk solution (30%) was used as a reinforcer (0.01 ml) and was delivered into a small cup by means of a dipper. Head entries into the food magazine were recorded by an infrared photo-cell beam crossing the entrance of the receptacle hole, which could be illuminated by a yellow stimulus light inside the aperture. A house-light was located at the top of the wall above the food magazine. The presentation of stimuli and the recording of the responses were controlled by a Smart Control Package 8IN/16out with an additional interface by MED-PC for Windows (Med Associates Inc., St. Albans, VT, USA).

### Experiment 1: Performance of Five-choice serial reaction time task by C57BL/6J and C57BL/6JOlaHsd mice

#### Habituation to the reinforcer and to the 5-CSRTT boxes

During the first three sessions animals were placed in the boxes for 30 min and the liquid reinforcer was available in the magazine on a continuous schedule. The animal had to nose-poke in the magazine to receive the condensed milk solution (available for 10 seconds), after which the dipper was refilled. The house-light, the magazine light and the stimulus lights in the five holes were turned on during the entire session. During these initial sessions, a drop of condensed milk was placed at the front of each of the nose-poke holes to encourage the mice to approach the holes and nose-poke in them. Magazine head entries and number of reinforcers earned were recorded. After these habituation sessions training in a non-spatial version of the 5-CSRTT began.

#### Non-spatial 5-CSRTT training

During the non-spatial training sessions the stimulus lights in the five holes were illuminated simultaneously and continuously and a nose-poke in any hole produced the illumination of the food magazine and was reinforced by the delivery of the condensed milk solution (procedure adapted from Patel *et al.* 2006). These sessions lasted until the animal earned more than 50 reinforcers during two consecutive days and for a maximum of 10 sessions, after which the animal was moved on to the spatial 5-CSRTT training.

#### Spatial 5-CSRTT training

The session started with the illumination of the house-light and a free delivery of the liquid reinforcer accompanied by the illumination of the food magazine, which signalled the availability of the reinforcer. When the animal nose-poked into the magazine to obtain the reinforcer (dipper on for 3 seconds.) the first trial was initiated. After a fixed interval (inter-trial interval, ITI) one of the stimulus lights in the holes was turned on for a brief time and the animal was required to nose-poke within a certain period of time (limited hold, LH) into the correct hole in order to earn the reinforcer. After a correct response the animal had to nose-poke into the magazine to collect the reward and to initiate the next trial. An incorrect response occurred when the animal made a response in a different hole to the one that had been illuminated, and this was followed by a time out (TO) period during which the lights were turned off for 5 seconds. Responses made into the holes during this period restarted the TO. An error of omission occurred when the animal failed to respond into any of the holes after the completion of the LH and was also followed by a TO. Any response into the holes during the ITI, which means that the stimulus light had not yet been presented, was registered as a premature response and was followed by a TO. After a TO period the next trial was restarted by a nose-poke into the magazine. Perseverative responses, that is, further responses into the holes after a correct response and before the collection of the reward, were registered but had no programmed consequences.

At the beginning of the training, the stimulus duration (SD) was set to 30 seconds and the ITI was set to 2 seconds, but these parameters were adjusted according to the performance of each animal. When the animal produced two consecutive sessions achieving the performance criteria (see Table 1) the SD was reduced in the following pattern: 30, 20, 10, 5, 2.5 and 1.8 seconds (Stage 6, baseline) and the LH and the ITI were set at 5 seconds. Testing was carried out daily (5–6 days a week), and sessions lasted for 100 trials or 30 min, whichever came first. On achieving criterion at Stage 6, animals were further trained for a minimum of seven sessions until stable responding was reached, and the average of the last three sessions under Stage 6 was used to establish baseline performance levels.

**Table 1:** Parameters used and criteria for progression during the successive stages of training in the 5-CSRTT (taken from Walker *et al.* 2011, with permission from the authors)

Training stage	Stimulus duration (seconds)	Limited hold (seconds)	Inter-trial interval (seconds)	Criteria for progression
1	30	30	2	>30 Correct trials
2	20	20	2	>30 Correct trials
3	10	10	5	>50 Correct trials
4	5	5	5	>50 Correct trials, >75% accuracy, <25% omissions
5	2.5	5	5	>50 Correct trials, >75% accuracy, <25% omissions
6	1.8	5	5	>50 Correct trials, >75% accuracy, <25% omissions

Following training under baseline conditions, mice were tested under conditions designed to increase premature responding. The effect of lengthening the ITI was tested: long ITI (10 seconds) sessions had a duration of 60 min. Long ITI challenge sessions were chosen because they have been shown to increase premature responding consistently in rats (Dalley *et al.* 2007) and mice (Oliver *et al.* 2009). Between each of the long ITI testing sessions mice performed a minimum of 3 days under baseline parameters (see Oliver *et al.* 2009; Walker *et al.* 2011 for further details of the 5-CSRTT protocol). The same procedure and time sequence was followed in all three 5-CSRTT experiments (except that in Experiment 2 a 2-month period elapsed between L1 and L2).

### Experiment 2: Performance of five-choice serial reaction time task by C57BL/6J WT and alpha-synuclein KO mice

Training was identical to Experiment 1.

### Experiment 3: Analysis of recombinant inbred strains

Male mice from 10 BXD recombinant inbred strains (see above) as well as C57BL/6J and DBA2/J parental strains were characterized in the 5-CSRTT. We used the %premature responding during baseline, and each of the three long ITI probe trials, to seek correlations with levels of snca expression in hippocampus, available from the WebQTL database (http://www.genenetwork.org/; Hippocampus consortium M430v2 (June 06) PDNN; Overall *et al.* 2009).

### Quantitative real-time PCR

Brains were removed and stored at −80°C prior to microdissection. Bilateral punches from the prefrontal cortex and hippocampus were taken and used for PCR analysis. RNA was extracted (Trizol) and cleaned (RNeasy MinElute, Qiagen, Hilden, Germany), cDNA was synthesized (iScript Select, Bio-Rad, Hercules, CA, USA) and subjected to quantitative RT-PCR (SYBR Green, Quanti-tect, Qiagen, Hilden, Germany) using the following primer pairs: alpha-synuclein, GATCCTGGCAGTGAGGCTTA and GCTTCAGGCTCATAGTCTTGG; beta-synuclein, GGAACCAGAAGGGGAAAGTT and CTCTGGCTCGTATTCCTGGT; gamma-synuclein, GCCAAAGAGCAAGAGGAGAA and TGTCCCTTGAGCCTCTGTG; GAPDH, TGTCTCCTGCGACTTCAAC and AGCCGTATTCATTGTCATACC. For each sample (run in triplicate) the cycle threshold was determined, and fold change of genes (from WT and Charles River) analysed according to the method of Pfaffl (Pfaffl 2001) and assessed by one-way analysis of variance (ANOVA).

### Statistical analysis

The statistical analysis was performed using the ‘Statistical Package for Social Sciences' (SPSS, version 18.0).

The variables considered in the analysis of the 5-CSRTT were

1. Accuracy (percentage of correct responses): correct responses/(correct responses + incorrect responses + omissions) × 100.
2. >Percentage of omissions: total omissions/(correct responses + incorrect responses + omissions) × 100.
3. Percentage of premature responding: premature responses/ (correct responses + incorrect responses + omissions + premature responses) × 100.
4. Correct latency: latency to nose-poke into the correct hole after the onset of the stimulus (s).
5. Magazine latency: latency to collect the reward after a correct response (s).
6. Perseverative responses: total number of responses made into the holes after a correct response and before the collection of the reward.

Two-way repeated measures ANOVA with strain or genotype as the between-subjects factor and session as the within-subjects factor was used for the analysis of each variable of the 5-CSRTT during the long ITI testing. Total number of sessions to achieve criteria of performance in the 5-CSRTT task was analysed by a non-parametric Kruskal-Wallis test. Independent samples *t* tests and paired samples *t* tests were used for *post hoc* analysis. Where sphericity assumptions were violated, the Greenhouse-Geisser correction was applied. The variables ‘accuracy’, ‘percentage omissions' and ‘premature responding’ were arcsine transformed [x′ = 2arcsine (√(*x*/100))] and ‘magazine latency’ and ‘perseverative responding’ were log_10 transformed in order to attain homogeneity of variance and permit valid parametric analysis. Level of gene expression data was analysed by one-way ANOVAs.

To investigate whether strain differences in premature responding may be attributable to strain differences in internal timing (or time estimation), we additionally analysed patterns of response timing from the onset of each trial. We argued that if premature responding is attributable to an overall tendency to perform at shorter time intervals, there would be a systematic shift in the pattern of responses to shorter time intervals. We thus analysed response latencies for all responses (including premature, correct and incorrect responses) by allocating responses to 1-second time bins, and performing ANOVA with the factors strain and time bin (repeated measure) for each long ITI session.

## Results

### Experiment 1. Comparison between C57BL/6J (C) and C57BL/6JOlaHsd (H) mice in the 5-CSRTT

**Figure 1:**
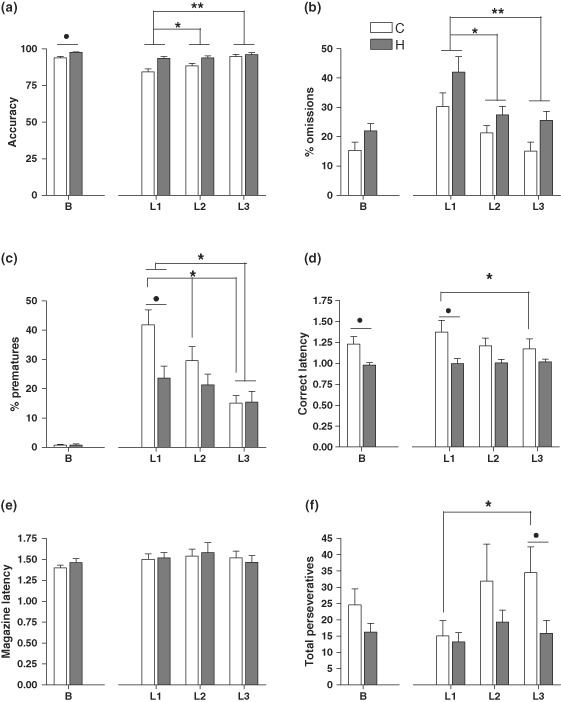
Performance of C57BL/ 6J mice from Charles River (C, clear bars) and C57BL/6JOlaHsd mice from Harlan (H, dark bars) in the 5-CSRTT for the baseline (B: mean of the last three sessions on Stage 6) and the three long ITI sessions (L1, L2, L3) The values represent the mean ± SE of accuracy of responding (a), percentage omissions (b), percentage of premature responses (c), latency to make a correct response (d), latency to collect the liquid reinforcer (e) and total perseverative responses (f). ^*^P < 0.05, ^**^P < 0.01, significant differences between sessions; 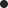
*P* < 0.05, significant differences between strains (*post hoc* tests).

Although there were no statistical differences in number of sessions required to achieve baseline performance criteria (H: 41.1 ± 2.87; C: 33.2 ± 2.6), a trend towards a significant effect of strain appeared (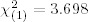, *P* = 0.054) suggesting that C mice needed fewer sessions than H mice to learn the task. H mice showed higher levels of accuracy (*t*_17_ = 3.894, *P* = 0.001) but a trend towards more omission errors (*t*_(17)_ = 1.913, *P* = 0.073) (see Fig. 1). No group differences appeared in impulsivity (%prematures), motivation (magazine latency) or compulsive responding (perseveratives) during baseline (*t <* 1.46, n.s.) but H mice showed significantly lower correct response latencies than C mice (*t*_17_ = 2.67, *P* < 0.05; see Fig. 1d).

Following the introduction of the long ITI sessions (see Fig. 1), H mice continued to display higher accuracy than C mice (*F*_1,17_ = 7.79, *P* < 0.05) but all mice improved accuracy as the long ITI sessions were repeated (*F*_2,34_ = 20.036, *P* < 0.001). At the same time, in keeping with their baseline performance, H mice consistently showed more omissions than C mice (*F*_1,17_ = 5.58, *P* < 0.05), although both groups decreased the percentage of omissions across the long ITI sessions (*F*_2,34_ = 21.332, *P* < 0.001). The increase in accuracy and the decrease in omissions in consecutive long ITI sessions indicated that the animals adapted successfully to the new parameters of the task.

With regard to the impulsivity measure (%premature responding) strain differences emerged upon the introduction of the long ITI sessions (*F*_2,34_ = 5.069, *P* < 0.05; Fig. 1c). As expected, all animals increased premature responding above the levels shown in baseline sessions (due to the longer waiting time imposed by the new parameters) and *post hoc* analysis indicated that during the first long ITI session H mice were less impulsive (fewer premature responses) than C mice. Premature responding was also decreased upon successive presentations of the long ITI sessions.

Also consistent with their baseline performance, H mice showed shorter correct response latencies than C mice (*F*_1,17_ = 5.57, *P* < 0.05; Fig. 1d), especially during the first long ITI session (session × group, *F*_2,34_ = 3.33, *P* < 0.05).

**Figure 3:**
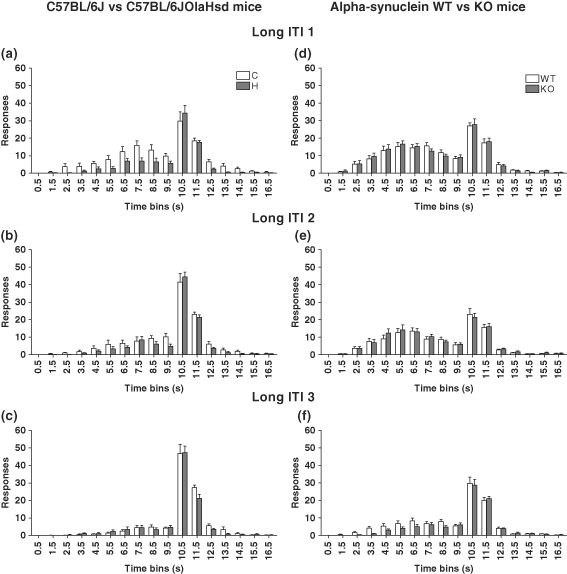
Distribution of total responses (correct + incorrect + premature) during the total time in which a response can be made for each trial (ITI: 10 seconds + SD: 1.8 seconds + LH: 5 seconds; total = 16.8 seconds) of the long ITI sessions (L1, L2 and L3) in the 5-CSRTT Figures a, b and c show the performance of C57BL/6J Charles River mice (C, clear bars) vs. C57BL/6JOlaHsd Harlan mice (H, dark bars) and d, e and f show the performance of alpha-synuclein wild type (WT, clear bars) vs. knock-out mice (KO, dark bars). ITI: inter-trial interval, SD: stimulus duration, LH: Limited hold (time allowed for nose-pose).

Analysis of the overall pattern of response times (Fig. 3) showed that C mice emitted more responses than H mice (*F*_1,17_ = 9.242, *P* < 0.01), and there was a significant variation across time bins (*F*_16,272_ = 46.381, *P* < 0.001) but there was no difference between strains in terms of the pattern of responding (no statistically significant interaction of time bin × group appeared in the analysis: *F*_16,272_ = 1.632, n.s.), suggesting no internal timing differences between groups in the first long ITI session. For the second and third long ITI sessions the main effect of group disappeared and only an effect of time bin remained (*F*_16,272_ > 114.27, *P* < 0.001 for L1 and L2). Especially on the first long ITI sessions (see Fig. 3a,d), the data follow a bimodal distribution: premature responses are distributed during the first 10 seconds, while correct and incorrect responses contribute to the values shown after the 10 seconds ITI, showing the highest values immediately after the stimulus is presented.

No differences between groups or sessions were found in motivation (magazine latency; *F <* 0.81, n.s.; Fig. 1e), but C mice showed an increase in compulsive (perseverative) responding from the first to the third long ITI session, whereas H mice showed similar levels across sessions and less perseveration than C mice in the third long ITI session (*F*_2,34_ = 3.985, *P* < 0.05; Fig. 1f).

### Experiment 2. Comparison of WT and alpha-synuclein KO mice in the 5-CSRTT

**Figure 2:**
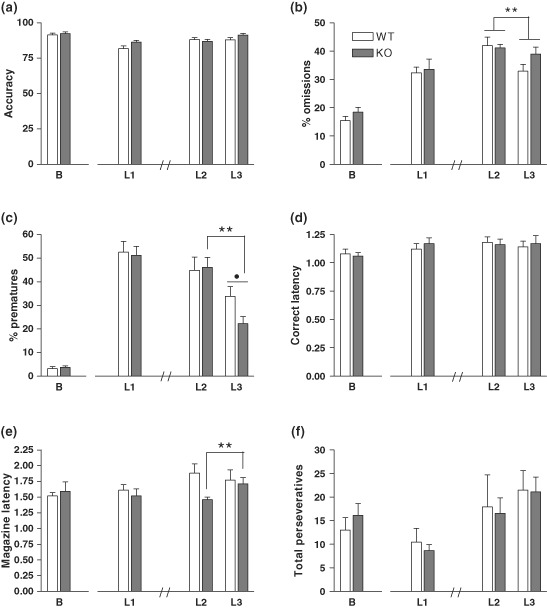
Performance of alpha-synuclein wild type (WT, clear bars) and knock-out (KO, dark bars) mice in the 5-CSRTT for the baseline (B: mean of the last three sessions on Stage 6) and the three long ITI sessions performed (L1, L2, L3) The values represent the mean ± SE of accuracy of responding (a), percentage omissions (b), percentage of premature responses (c), latency to make a correct response (d), latency to collect the liquid reinforcer (e) and total perseverative responses (f). ^*^p < 0.05, ^**^p < 0.01, significant differences between sessions; 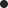
*P* < 0.05, significant differences between strains (*post hoc* tests).

Mice did not differ in acquisition of the task as indicated by the similar number of training sessions to achieve performance criteria (WT: 36.87 ± 3.28; KO: 34.69 ± 2.76, 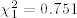, n.s.,). No statistical differences between groups were found in any of the variables measured during baseline (see Fig. 2, all *t <* 1.53, n.s.).

Figure 2 also shows the data from the three long ITI sessions. A 2-month period elapsed between the first long ITI (L1) and the second (L2); consequently two separate statistical analyses were performed. During the first long ITI session no statistical differences between groups emerged in any variable (all *t <* 1.9, n.s.). Analysis of the second and third long ITI sessions showed that both groups presented equal levels of accuracy and of percentage of omissions, although, during the third long ITI session both groups showed a decrease in omissions in comparison with the second long ITI (see Fig. 2b).

With regard to impulsivity, the analysis revealed a session × group interaction in percentage of premature responding (*F*_1,29_ = 4.86, *P* < 0.05; Fig. 2c) due to a decrease in premature responding in snca KO mice compared to WT. *Post hoc* comparisons showed a significant decrease in premature responding in the third long ITI session compared to Session 2 in KO, but not WT mice (KO = *t*_15_ = 5.12, *P* < 0.0001); (WT = *t*_14_ = 1.78, n.s.) and that KO mice were significantly less impulsive than WT during the last long ITI session (*t*_30_ = 17.15, *P* < 0.0001). As for magazine latencies, a session × genotype interaction appeared in the analysis (*F*_1,29_ = 45.16, *P* < 0.05), indicating faster latencies in KO mice during L2 but no differences between groups in L3. No statistical differences in correct latency or perseverative responses (all *F*_1,29_ < 2.87, n.s.) were found.

Analysis of the overall pattern of response times in L1, L2 and L3 showed no statistically significant interaction between strain and time bin, suggesting that the decrease in premature responses seen in *snca* KO mice during L3 was not due to a different timing of responses. Only an effect of ‘time bin’ was found in the repeated measures ANOVA (*F*_6,512_ > 45.63, *P* < 0.001, for L1, L2 and L3; see Fig. 3) which is explained by the differential contribution of premature responses (before 10 seconds) and correct and incorrect responses (from 10 seconds onwards) along the *x* axis.

### Experiment 3. Recombinant inbred strains

**Figure 4:**
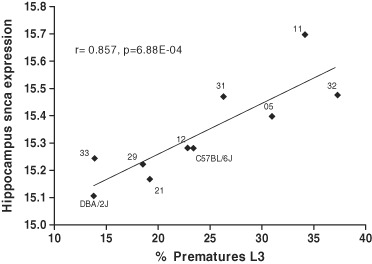
Correlation of mRNA levels of alpha-synuclein in hippocampus (*y* axis) and percentage of premature responding in the third long ITI session (L3, *x* axis) for DBA2/J, C57BL/6J and BXD5, 11, 12, 21, 29, 31, 32 and 33 strains (no mRNA data available for BXD18 and BXD36 strains) A highly significant positive correlation between level of expression of alpha-synuclein in hippocampus and percentage of premature responding was seen (*r* = 0.857, *P* = 0.00069).

As loss of alpha-synuclein was associated with lower %premature responding, we tested the hypothesis that variation in impulsivity across 10 recombinant inbred BXD and parental strains would be associated with expression levels of snca. Premature responding during baseline conditions (*R* = 0.679; *P* = 0.028), as well as during the first (*R* = 0.713; *P* = 0.0188) and third (*R* = 0.857; *P* = 0.00068, see Fig. 4) long ITI correlated positively with alpha-synuclein mRNA levels in hippocampus (Overall *et al.* 2009), an area of high alpha-synuclein expression (Vivacqua *et al.* 2011).

### Quantitative real-time PCR

**Figure 5:**
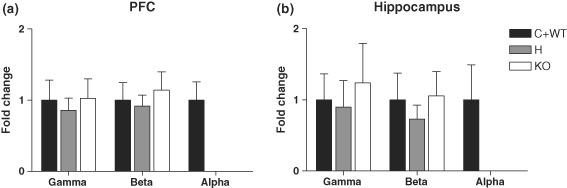
mRNA quantification of gene expression by means of quantitative RT-PCR Messenger RNA levels were normalized against GAPDH. Gene expression of gamma, beta and alpha-synuclein in the Prefrontal Cortex (PFC) (a) and Hippocampus (b) is shown as fold change in expression of Harlan (H) and alpha-synuclein knock-out mice (KO) vs. combined data of WT+Charles mice (±SEM).

No statistical differences in gene expression of gamma- and beta-synuclein in H and KO groups with comparison to the WT appeared in the analysis, indicating no differences in compensation following loss of snca in KO and H mice (Fig. 3; *F <* 1.6, n.s.). An effect of group on expression in PFC (*F*_2,22_ = 482.7, *P* < 0.0001, Fig. 5a) and hippocampus (*F*_2,23_ = 540.4, *P* < 0.0001, Fig. 5b) confirmed the absence of expression of snca in H and KO mice.

## Discussion

The present experiments reveal a role for alpha-synuclein in ‘waiting’ impulsivity, based on three pieces of evidence. In Experiment 1, H mice, with a spontaneous deletion of a section of chromosome 6 leading to loss of the snca gene encoding alpha-synuclein, showed lower impulsivity than its ancestral C strain when required to withhold a pre-potent response in the 5-CSRTT. Although both strains showed high levels of accuracy of responding throughout the experiment, in both baseline and challenge conditions H mice performed significantly more accurately than C animals. Together with this superior accuracy, H mice generally showed more omissions (i.e. less readiness to respond) than C mice. This effect appeared during each of the long ITI sessions, suggesting a different strategic approach to the task in the two sub-strains. We can speculate that H mice adopted a more conservative or inhibited approach, which might be described by a rule ‘when uncertain, do not respond, but when certain respond quickly’ resulting in more omissions, but higher accuracy and shorter correct latencies. The observation that H mice showed higher speed of responding (shorter correct latencies) rules out general motor impairment or a diminished behavioural activation in this strain. Following the same argument, the higher level of impulsive (premature) responses in C mice was not due to a greater readiness to respond because they showed slower correct latencies than H mice. At the same time, the differences between sub-strains in their latencies to make a correct response were not due to differences in general responding because latencies of incorrect and premature responses did not differ significantly between groups (data not shown) suggesting that this effect is specific to correct responses.

A potential explanation of increased premature responding might include differences in internal time perception (see Sanchez-Roige *et al.* in press, for further discussion of the potential contribution of timing to impulsivity in the task). To address this possibility, we analysed patterns of responding (including correct, incorrect and premature responses) during the total time allowed in each trial; differences in time estimation would result in time-shifts of all responses. No evidence was found of altered timing behaviour between C57BL/6J and C57BL/6JOlaHsd strains. It thus seems unlikely that the differences we observed in premature responding are attributable to differences in internal timing between strains.

C57BL/6J mice thus appear to have difficulties in waiting to obtain the information necessary for accurate responding, or show more inflexibility in the switch from baseline to long ITI sessions. It is unlikely that differences in motivation to obtain the reinforcer contribute to differences in impulsive responding as magazine latencies did not differ between groups either for the baseline or long ITI conditions. With regard to perseverative responding, C mice showed generally higher levels than H mice and their perseveration increased across successive sessions as compared with the more stable pattern of responses in the H mice. However, because the chromosomal deletion resulted in the loss of genetic material in addition to the snca gene, these findings cannot be viewed as conclusive, even though multimerin1 is not expressed in the CNS (Specht & Schoepfer 2004), and is therefore unlikely to have contributed to the impulsive phenotype.

In the second experiment, WT and snca KO mice did not show differences in any of the variables measured in the training phases of the 5-CSRTT. However, like H mice, KO mice showed lower levels of premature responding than WT mice during the third long ITI session. Together these findings support the hypothesis that alpha-synuclein is involved in the regulation of impulsivity. Again, analysis of response timing failed to show differences in the pattern of responses between WT and KO mice, indicating that the differences in premature responses during L3 were not due to altered internal timing in the mutant mice.

Despite similarities in the two experiments, in Experiment 2 differences in impulsivity between groups did not appear until the third long ITI session. An informal comparison of performance during baseline conditions between the two experiments finds reduced levels of premature responding in Experiment 1 in comparison with Experiment 2 (see Figs. 1c and 2c), and equally, in the first long ITI session, premature responses were higher in Experiment 2. Thus, baseline differences in the two experiments could in principle account for the delay in the appearance of the less impulsive phenotype in KO mice in comparison with H mice. With regard to perseverative responding, a closer examination of the values during baseline finds higher values in C mice in comparison to H mice, which in turn resemble WT and KO mice.

Another potential explanation for the differences associated with loss of the snca gene in the two experiments could be different compensations within the synuclein family resulting from the different extents of the deletions in the mice. The chromosomal deletion in the H mice results in the loss of the coding regions of both the snca and mmrn1 genes together with regulatory regions of these, and potentially other genes (Specht & Schoepfer 2001, 2004), and unidentified microRNA-encoding sequences that might trans-regulate expression of other genes. In contrast, the snca KO mouse has been created by the deletion of the first two exons of the gene, encoding amino acids 1–41 and upstream untranslated regions (Abeliovich *et al.* 2000). The more localized disruption of the snca gene in the KO mice could trigger different compensations within the synuclein family compared to those taking place in the H mice, which could contribute to the differences in the two experiments. The synucleins are a family of three members, alpha-, beta- and gamma-synuclein that share considerable N-terminal amino acid sequence homology (Jakes *et al.* 1994; Nakajo *et al.* 1993). When one or more synucleins is deleted, the remaining members of the family are upregulated in some brain regions (Chandra *et al.* 2004; Robertson *et al.* 2004). That similar changes in gene regulation occur in response to targeted deletion of alpha- or gamma-synuclein, suggests a degree of functional overlap (Kuhn *et al.* 2007). We addressed this issue by investigating levels of expression of all three members of the synuclein family in prefrontal cortex and hippocampus of C and WT strains, as well as H and KO mice. We found no evidence for differential compensations at the gene expression level of gamma- or beta-synuclein in H or KO mice in comparison with the wild-types, suggesting that differences between H and KO mice are not attributable to differences in compensatory mechanisms within the synuclein family in prefrontal cortex or hippocampus. Nevertheless, the possibility of differential compensations within other brain areas such as the striatum (dorsal/ventral), or at a protein level, cannot be ruled out.

Third, we compared levels of impulsive responding across a series of mouse strains from the BXD series of recombinant inbred strains, and correlated levels of impulsivity with levels of expression of the snca gene in hippocampus, a brain area in which snca expression is prominent (Vivacqua *et al.* 2011), and which has been implicated in impulsive behaviour (Urcelay & Dalley 2011). Remarkably, the level of snca expression was associated with premature responding under baseline as well as long ITI conditions. Specifically, the correlation between snca expression in hippocampus and impulsivity levels was especially significant during the third long ITI session, which accounted for 73% of the variation in L3 impulsivity. While such correlative evidence can only be suggestive, taken with the convergent findings from Experiments 1 and 2, these results implicate snca expression in 5-CSRTT impulsivity.

Although the normal biological functions of alpha-synuclein are not well understood, it plays a role in synaptic vesicular transport and synaptic plasticity (Burre *et al.* 2010; Greten-Harrison *et al.* 2010; Murphy *et al.* 2000; Nemani *et al.* 2010). Mice lacking alpha-synuclein show alterations in dopaminergic neurotransmission in striatal areas (Abeliovich *et al.* 2000; Anwar *et al.* 2011; Nemani *et al.* 2010; Senior *et al.* 2008; Venda *et al.* 2010). Possible changes in dopamine function as a result of the deletion of the snca gene might give rise to the low impulsive phenotype seen in H and KO mice in the present investigation. In line with this, rats selected for high impulsivity in the 5-CSRTT show low levels of dopamine D2/3 receptors in ventral striatum (Dalley *et al.* 2007), and PD patients with a pathological gambling disorder had lower D2/3 receptor binding in ventral striatum (Steeves *et al.* 2009).

PD patients have been suggested to possess a premorbid low impulsive personality (Dagher & Robbins 2009; Ishihara & Brayne 2006; Menza 2000; Paulson & Dadmehr 1991; Todes & Lees 1985). Following manifestation of the condition, an association between PD and ICDs has been increasingly reported. These include repetitive, excessive and compulsive activities that interfere with normal life (Evans *et al.* 2005, 2009; Lawrence *et al.* 2007), manifested as pathological gambling, compulsive shopping, punding (repetitive purposeless behaviours) and hypersexuality, that appear in PD patients after dopamine replacement therapy (For reviews see Evans *et al.* 2009; Voon *et al.* 2007, 2009). As not all patients taking dopaminergic agonist treatment develop ICD, it has been speculated that dopaminergic medication might trigger ICD in individuals with specific impulsivity traits (Isaias *et al.* 2008; Voon *et al.* 2007, 2009). The results from the present investigation suggest that it is worth investigating whether alpha-synuclein may contribute to these differences in impulsivity present in PD patients, possibly through its modulation of the dopaminergic system (Dagher & Robbins 2009; Venda *et al.* 2010). Whether differences in levels of expression, or haplotypic variations in the gene encoding alpha-synuclein contribute to the propensity to develop ICDs remains to be investigated.
